# AICAR inhibits adipocyte differentiation in 3T3L1 and restores metabolic alterations in diet-induced obesity mice model

**DOI:** 10.1186/1743-7075-3-31

**Published:** 2006-08-10

**Authors:** Shailendra Giri, Ramandeep Rattan, Ehtishamul Haq, Mushfiquddin Khan, Rifat Yasmin, Je-song Won, Lyndon Key, Avtar K Singh, Inderjit Singh

**Affiliations:** 1 Department of Pediatrics, Medical University of South Carolina, Charleston, SC 29425, USA; 2 Department of Pathology, Medical University of South Carolina, Charleston, SC 29425, USA; 3 Division of Experimental Pathology, Department of Laboratory Medicine and Pathology, Mayo Clinic/Foundation, 200 First Street, SW Rochester, MN 55905, USA; 4 Department of Pathology and Laboratory Medicine, Ralph Johnson Veterans Affairs Medical Center, Charleston, SC 29425, USA

## Abstract

**Background:**

Obesity is one of the principal causative factors involved in the development of metabolic syndrome. AMP-activated protein kinase (AMPK) is an energy sensor that regulates cellular metabolism. The role of AMP-activated protein kinase in adipocyte differentiation is not completely understood, therefore, we examined the effect of 5-aminoimidazole-4-carboxamide-1-β-D-ribofuranoside (AICAR), a pharmacological activator of AMP-activated protein kinase (AMPK) on adipocyte differentiation in 3T3L1 cells and in a mouse Diet induced obesity (DIO) model.

**Methods:**

To examine the effect of AICAR on adipocyte differentiation in 3T3L1 cells and in a mouse Diet induced obesity (DIO) model, 3T3L1 cells were differentiatied in the presence or absence of different concentration of AICAR and neutral lipid content and expression of various adipocyte-specific transcription factors were examined. *In vivo *study, treated and untreated mice with AICAR (0.1–0.5 mg/g body weight) were fed high-fat diet (60% kcal% fat) to induce DIO and several parameters were studied.

**Results:**

AICAR blocked adipogenic conversion in 3T3L1 cells along with significant decrease in the neutral lipid content by downregulating several adipocyte-specific transcription factors including peroxisome proliferators-activated receptor γ (PPARγ), C/EBPα and ADD1/SREBP1, which are critical for adipogenesis *in vitro*. Moreover, intraperitoneal administration of AICAR (0.5 mg g/body weight) to mice fed with high-fat diet (60% kcal% fat) to induce DIO, significantly blocked the body weight gain and total content of epididymal fat in these mice over a period of 6 weeks. AICAR treatment also restored normal adipokine levels and resulted in significant improvement in glucose tolerance and insulin sensitivity. The reduction in adipose tissue content in AICAR treated DIO mice was due to reduction in lipid accumulation in the pre-existing adipocytes. However, no change was observed in the expression of PPARγ, C/EBPα and ADD1/SREBP1 transcription factors *in vivo *though PGC1α expression was significantly induced.

**Conclusion:**

This study suggests that AICAR inhibits adipocyte differentiation via downregulation of expression of adipogenic factors *in vitro *and reduces adipose tissue content in DIO mice by activating expression of PGC1α without inhibiting adipocyte-specific transcription factors in DIO mice.

## Background

The intake of calorie-rich fast food and sedentary lifestyles of developed countries has sharply increased the incidence of obesity. Obesity is not only a serious health and economic burden, but also predisposes a person to a variety of metabolic diseases (i.e., the coexistence of several risk factors for atherosclerosis, hyperglycemia, dyslipidemia, and hypertension), where the mechanistic role of obesity is yet to be fully elucidated. Obesity is simply the consequence of an imbalance between energy intake and expenditure by the body from a metabolic point of view. The main sites of energy utilization, skeletal muscle and adipose tissue play significant roles in the regulation of energy homeostasis. Skeletal muscles are one of the major organs responsible for insulin-mediated glucose disposal, and maintenance of glucose homeostasis of the body. Adipose tissue serves as an energy storage depot to maintain lipid homeostasis, thereby promoting the survival ability of the human body [1]. Obesity occurs when the adipose tissue is overloaded with high-energy nutrients without subsequent expenditure.

Because adipocytes play a critical role in energy balance, understanding the molecular mechanisms of adipocyte differentiation may provide clues for developing strategies for the prevention and treatment of obesity. The mechanisms of adipocyte differentiation have been extensively studied in preadipocyte culture systems. Characterization of the regulatory regions of adipose-specific genes has led to the identification of key transcription factors in the complex transcriptional cascade that occurs during adipocyte differentiation [2]; these factors include peroxisome proliferator-activated receptor γ (PPARγ) [3], CCAAT/enhancer binding protein (C/EBP) [4,5], adipocytedifferentiation and determination factor 1(ADD1), and sterolregulatory element binding protein 1c (SREBP1c) [2,6-8].

In addition to functioning as the principal energy storage depot, adipose tissue secretes hormones (adipokines) that contribute to energy homeostasis [9]. One such hormone, leptin, acts chiefly through the brain to regulate food intake and energy expenditure. Other adipokines, including tumor necrosis factor-α (TNFα), adiponectin, adipsin, and interleukin-6 (IL-6) can also regulate the inflammatory response [10-13]. The list of cytokines identified in white adipose tissue (WAT) is increasing, suggesting that obesity also results in a chronic, low level of inflammation through production of these proteins [11-15]. This inflammatory state may contribute to major obesity associated complications, including type 2 diabetes, cardiovascular disease, and the metabolic syndrome. Also, adipose TNFα expression is increased in obesity and has been shown to contribute towards insulin resistance [11].

The AMP-activated protein kinase (AMPK) is an evolutionarily conserved sensor of cellular energy status, and plays a critical role in systemic energy balance in response to metabolic stress [16-18]. AMPK is a serine/threonine protein kinase and a member of the Snf1/AMPK protein kinase family [16-18]. It is a αβγ heterotrimeric protein, consisting of a α catalytic subunit, a β subunit important both for enzyme activity and for targeting, and a γ regulatory subunit, which binds the allosteric activator, AMP. The activity of AMPK absolutely requires phosphorylation of the α subunit on Thr-172 in its activation loop by one or more upstream kinases (AMPKK) [16]. AMPK integrates nutritional and hormonal signals in peripheral tissues with the hypothalamus and also mediates adipokine effects (leptin, adiponectin, and possibly resistin) in regulating food intake, body weight and glucose and lipid homeostasis. Recently, CaMKKs and LKB have been identified as its upstream kinase, AMPKK [19-21]. 5-Aminoimidazole-4-carboxamide-1-β-D-ribofuranoside (AICAR) is a known activator of AMPK and has been used as an experimental tool to activate AMPK *in vitro *and *in vivo*. It has been shown that chronic administration of AICAR in rats resulted in marked changes in skeletal muscle that included increases in glycogen stores and GLUT4, and increased activity of hexokinase and mitochondria oxidative enzymes [22-24]. Administration of AICAR also led to an increase in maximal insulin-stimulated glucose transport and GLUT4 translocation [25]. Altogether it is conceivable that repetitive activation of AMPK may be part of the mechanism leading to improved insulin action after exercise.

In the present study, we examined the effect of AICAR on adipocyte differentiation using an *in vitro *model, i.e. 3T3L1 cell line, and an *in vivo *diet induced obesity mouse model.

## Methods

### Reagents

AICAR was purchased from Toronto Chemicals. Antibodies against phosphospecific as well as nonphospho-AMPKα and ACC were from Cell Signaling (Beverly, MA). Antibodies against AMPK α1 and α2 were from Abcam (Cambridge, MA). Antibodies against C/EBPα, C/EBPβ, SREBP1, PPARγ, β actin and proteinA/G plus agarose were from Santa Cruz Biotechnology (Santa Cruz, CA). [^3^H]Thymidine was from PerkinElmer Life Sciences (Boston, MA). 5-iodotubercidin was purchased from Calbiochem (San Diego, CA).

### 3T3-L1 cell culture and differentiation

3T3-L1 preadipocytes were cultured in Dulbecco's modified Eagle's medium (DMEM) containing 10 % (v/v) NBS. AICAR was dissolved in DMSO for cell culture studies (0.5 M, stock). On day 0 (2 day after 3T3-L1 preadipocytes reached confluence), cells were induced to differentiate by insulin (1.7 μmol/L), IBMX (0.5 mmol/L), and DEX (1 μmol/L). On day 3 and every other 3^rd ^day thereafter, fresh regular medium was substituted until day 9 [19]. Differentiation was monitored by morphological assessment and Oil red O staining [20]. For Oil red O staining, cells were washed twice with PBS, fixed in 3.7 % formaldehyde for 1 h, and stained for 30 min with 0.2 % (w/v) oil red O solution in 60 % (v/v) isopropanol. Cells were then washed several times with water, and excess water was evaporated by placing the stained cultures at approximately 32°C. In order to determine the extent of adipose conversion, 0.2 ml of isopropanol was added to the stained culture dish. The extracted dye was immediately removed by gentle pipetting and its optical density was monitored spectrophotometrically at 510 nm. For cell proliferation, 3T3L1 cell were kept in differentiating media in the presence of AICAR/iodotubercidin (0.5 mM/0.1 μM) for three days. [^3^H] thymidine (1 μCi/well) was added at the end of the treatment for 6 h and mean incorporation of thymidine in DNA was measured by a 1450 Microbeta Wallac Trilux Liquid Scintillation Counter (Perkin-Elmer Life Sciences) as described before [26].

### Mice

Male C57BL/6 mice were purchased from The Jackson Laboratory (Bar Harbor, ME). Mice were fed a high-fat diet (60% kcal% fat) (Research Diets, New Brunswick, NJ) ad libitum for 6 weeks. At 1, 2, 4 and 6 weeks, animals were sacrificed and various tissues harvested, weighed, and processed for subsequent analysis. All procedures were performed in accordance with the experimental guidelines for animal care and use at the Medical University of South Carolina (Charleston, SC, USA).

### Administration of AICAR

To study the effect of AICAR, five groups of mice were allocated into group 1: mice fed with standard diet (STD); group 2: STD mice treated with AICAR (0.5 mg/g bw); group 3: mice fed high fat diet (DIO); group 4: DIO mice treated with AICAR (0.1 mg/g bw); group 5: DIO mice treated with AICAR (0.5 mg/g bw). The mice were treated with AICAR (0.1 or 0.5 mg/g body weight, dissolved in PBS, i.p. injection volume of 200 μl/mouse) three times a week for 6 weeks (Monday, Wednesday and Friday). Mice in the control group received 200 μl of PBS.

### Histology and cell-size measurement

Freshly isolated epididymal white adipose tissue from treated and untreated mice was fixed in 10% formalin and embedded in paraffin. Sections (8 μm) were stained with hematoxylin and eosin, and cell morphology and size were analyzed.

### RNA extraction, Northern blot analysis, and real-time PCR

After extraction of total RNA from white adipose tissue using of TRIzol (Invitrogen) as per the manufacturer's protocol, total RNA (5 μg) was used to prepare cDNA using iScript cDNA Synthesis Kit (BioRad Laboratories). Real-time PCR was performed as described previously utilizing Bio-Rad iCycler (iCycler iQ Multi-Color Real-Time PCR Detection System) using iQ SYBR Green Supermix reagent as per the instructions from BioRad Laboratories. The primer sets for use were designed (Oligoperfect Designer, Invitrogen) and synthesized from Integrated DNA Technologies. The primer sequences for PPARγ, SREBP1, C/EBPα, aP2, Adipsin and 18S taken from Le Lay *et al *[27]. Primer sequences are follows: DGAT1 for, 5'-TTCCGCCTCTGGGCATT-3'; rev, 5'-AGAATCGGCCCACAATCCA-3'; GPAT for, 5'-ATTGACACCTGCTGCTTTTGA-3'; rev, 5'-CCTACCCACTACAACAGAG-3'. Thermal cycling conditions were as follows: activation of DNA polymerase at 95°C for 10 min, followed by 40 cycles of amplification at 95°C for 30 sec and at 58.3°C for 30 sec. The normalized expression of target gene with respect to 18S was computed for all samples by using the Microsoft Excel data spreadsheet.

### AMPKα1 activity

AMPKα1 activity was assayed in isolated epididymal white adipose tissue from different groups using anti-AMPKα1 antibody for immunoprecipitation. For this, isolated epididymal white adipose tissues were homogenized in lysis buffer (50 mM Tris-HCl, pH 7.4, containing 50 mM NaCl, 1 mM EDTA, 0.5 mM EGTA, 10% glycerol, 1% triton X-100 and protease inhibitor mixture). Approximately 200 μg of tissue homogenate was incubated with anti-AMPKα1 antibody for 2 hr, then 30 μl of protein A/G plus agarose was added and incubated for an additional 1 hr at 4°C. The immune complexes were washed twice in lysis buffer and twice in kinase buffer (62.5 mM HEPES, pH 7.0, 62.5 mM NaCl, 62.5 mM NaF, 6.25 mM sodium pyrophosphate, 1.25 mM EDTA, 1.25 mM EGTA, and 1 mM dithiothreitol), incubated at 30°C in 30 μl of kinase assay buffer containing 200 μM AMP, ATP mixture (200 μM ATP and 1.5 μCi of [γ-^32^P]ATP), with or without 250 μM SAMS peptide (HMRSAMSGLHLVKRR) for 20 min. The reaction was terminated by spotting the reaction mixture on phosphocellulose paper (P81), and the paper was extensively washed with 150 mM phosphoric acid. The radioactivity was measured with a scintillation counter as described before [28].

### Tissue lipid content and fatty acid composition

Lipids were extracted by the Folch method in a mixture of 2:1 chloroform/methanol (vol/vol). The extract was washed with 0.2 volumes of saline (NaCl 0.9%) and centrifuged at 2,000 rpm for 10 min. The organic phase was recovered and serum triglycerides, cholesterol and free fatty acids (FFAs) were analyzed using HPTLC as described earlier [29]. Fatty acid composition of adipose tissue was determined using GC17A and capillary column as described [30].

### Metabolic measurements

Blood glucose was measured with a glucometer (AccuChek; Roche Diagnostics, Indianapolis, IN). Serum adiponectin and leptin concentrations were determined using a mouse adiponectin and mouse leptin enzyme-linked immunosorbent assay kits (BioVendor Laboratory Medicine, Inc.), respectively.

### Glucose and insulin tolerance tests

A glucose tolerance test was performed on mice fasted for 12 h. Blood glucose levels were determined at 0, 15, 30, 60, and 120 min after an intraperitoneal injection of glucose (2 g/kg body wt). For the insulin tolerance test, animals fasted for 5 h were injected intraperitoneally with 0.5 units insulin/kg body wt (sigma) and glucose levels were measured at 0, 15, 30, 60, and 120 min postinjection.

### Statistical analysis

Results shown represent means ± SD. Statistical analysis was performed by ANOVA by the Student-Neumann-Keuls test using GraphPad InStat software (San Diego, CA).

## Results

### AICAR inhibits differentiation of preadipocytes

**Figure 1:**
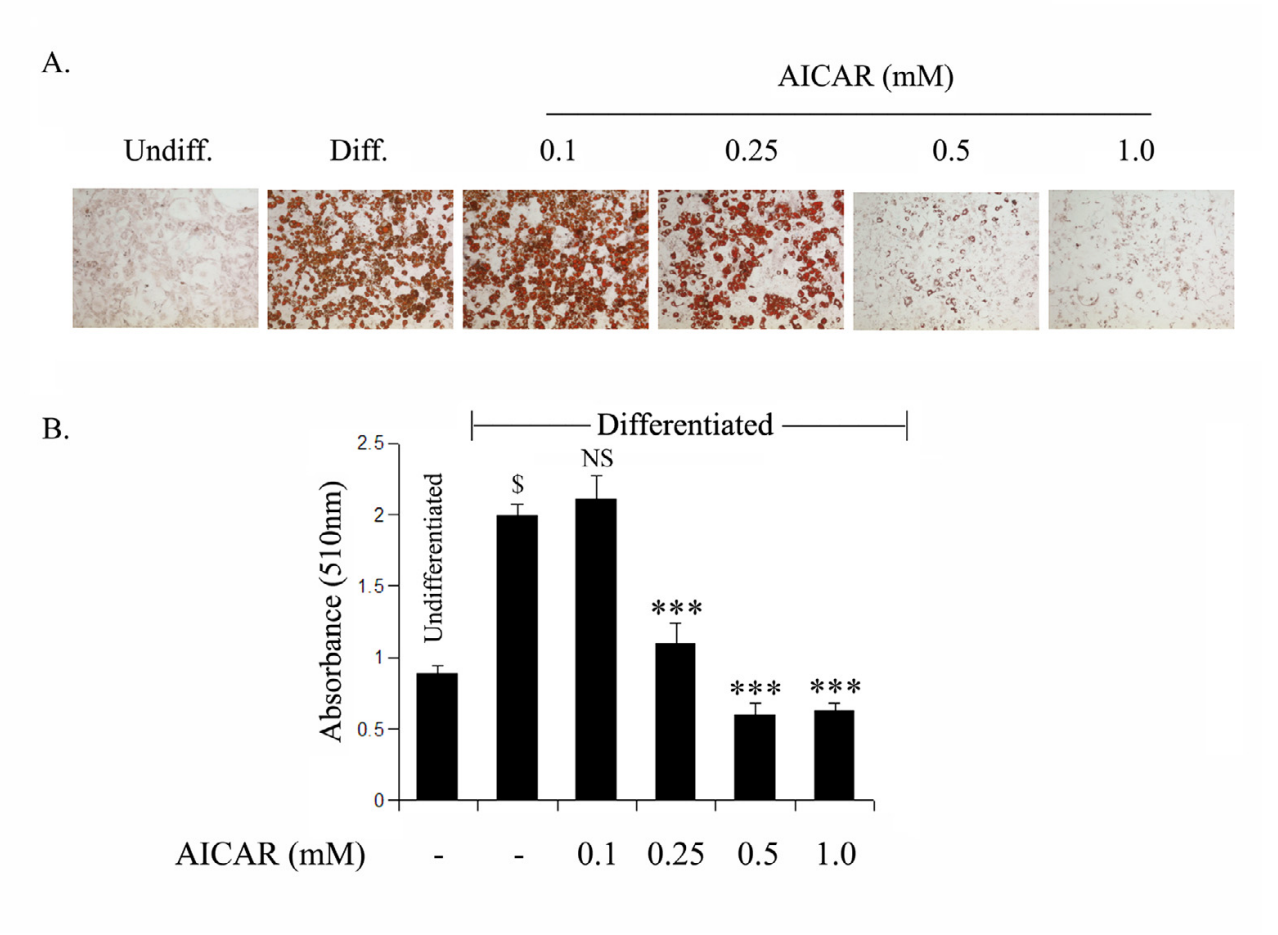
**AICAR inhibits 3T3L1 adipocyte differentiation in a dose dependent manner**. The differentiating 3T3-L1 cells were treated with AICAR at various concentrations for 9 days (from day 0 to day 9). Intracellular lipid was stained with Oil Red O (a). To determine the extent of adipose conversion, 0.2 ml of isopropanol was added to the 12 well plates. The extracted dye was immediately removed and its optical density was monitored spectrophotometrically at 510 nm (b). Results are the mean ± SD of three determinations. $, *p *< 0.001 as compared with untreated cells; NS, not significant; ***, *p *< 0.001 as compared with untreated differentiated sample.

We investigated the effect of AICAR on the induction of terminal differentiation markers at end of the differentiation period (day 9). Oil Red O staining showed that untreated differentiated cells had many lipid droplets indicating lipid accumulation. However, lipid accumulation was inhibited by AICAR treatment in a dose dependent manner (Fig. 1a). This observation was further supported with the quantitative analysis of neutral lipid content by measuring the absorbance at 510 nM (Fig. 1b). Untreated and AICAR treated (0.1 mM) differentiated 3T3L1 cells showed higher levels of lipid staining with the Oil Red O dye, which was significantly reduced by higher doses of AICAR (0.25–1.0 mM) treatment (Fig. 1b).

**Figure 2:**
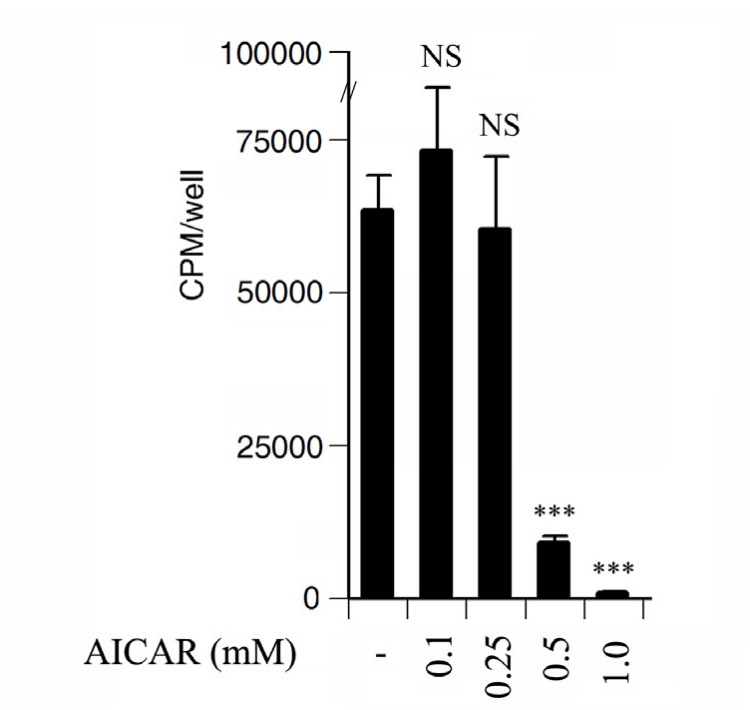
**Effect of AICAR treatment on cell proliferation during the 3T3L1 preadipocyte differentiation induction process**. 3T3-L1 cells were treated with the indicated concentrations of AICAR (from 0.1 to 1.0 mM) during the differentiation induction phase (from day 0 to day2) followed by [3H] thymidine uptake to examine cell proliferation. Results are the mean ± SD of three determinations. NS not significant; *** *p *< 0.001 as compared with untreated cells.

To examine further the effect of AICAR on cell proliferation, undifferentiated 3T3L1 cells were treated with increasing concentrations of AICAR (0.1–1.0 mM) in proliferative media for 3 days and cell proliferation was examined using [H3] thymidine uptake. AICAR at low concentrations (0.1 and 0.25 mM) did not have any effect on 3T3L1 cell proliferation but at higher concentrations (0.5–1 mM) it completely blocked cell proliferation (Fig. 2).

### AICAR inhibits key adipogenic transcription factors and markers

**Figure 3:**
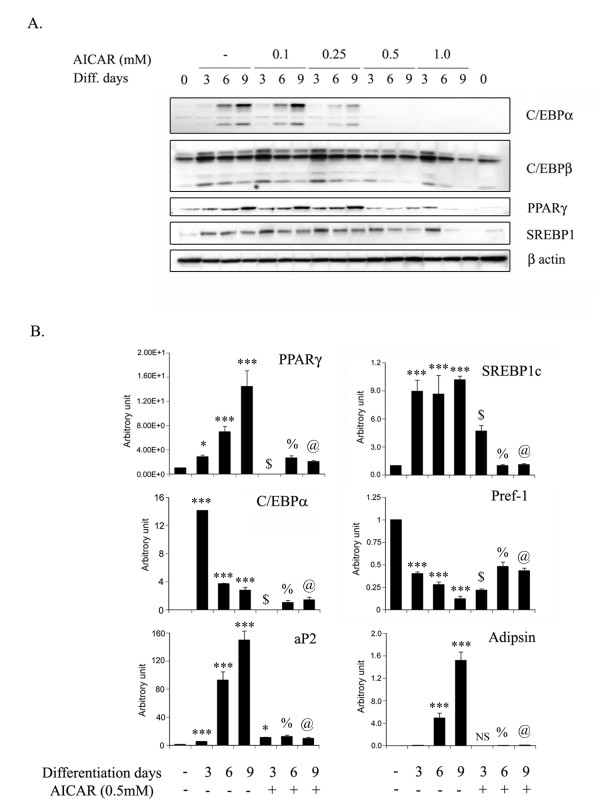
**Effect of AICAR on the expression of key adipogenic transcription factors during 3T3L1 adipocyte differentiation**. The differentiating 3T3-L1 cells were treated with different concentrations of AICAR (from 0.1 to 1.0 mM) and proteins were isolated at various time periods (from 0 to 9 days). Immunoblot analysis for various key adipogenic transcription factors including C/EBPα, C/EBPβ, PPARγ and SREBP1 were examined as described in Materials and Methods (a). The expression levels of PPARγ, SREBP1, C/EBPα, Pref-1, aP2 and adipsin mRNA were estimated by quantitative PCR (qPCR) using their specific primers as described in Materials and Methods (b). The relative qPCR values were normalized with respect to 18S rRNA expression levels. Values are mean ± S.D. of three determinations. *P < 0.05, *** *p *< 0.001 as compared with untreated cells. NS not significant; *p < 0.05, $ *p *< 0.001 as compared to 3 days differentiated samples. % *p *< 0.001 as compared to 6 days differentiated samples. @ *p *< 0.001 as compared to 9 days differentiated samples.

Adipogenesis is a highly regulated process requiring coordinated expression and activation of key transcription factors which include CCAAT/enhancer binding proteins (C/EBPs), peroxisome proliferators activated receptor gamma (PPARγ), and sterol regulatory element-binding proteins (SREBPs). To identify the molecular mechanism for the inhibition of adipocyte differentiation by AICAR treatment, the protein expression of key transcriptional factors for adipocyte differentiation was examined (Fig. 3a). The levels of C/EBPα protein expression in 3T3L1 during differentiation increased at day 6 and 9, which was inhibited by AICAR treatment in a dose dependent manner (Fig. 3a). However, the expression of C/EBPβ levels was unaffected by AICAR treatment. The expression of PPARγ and SREBP-1 was also significantly inhibited by AICAR treatment at 0.5 to 1 mM concentration (Fig. 3a). To support further these observations, the mRNA levels of these transcription factors were examined by quantitative analysis using real time PCR. As depicted in figure 3b, the mRNA expression of C/EBPα, PPARγ and SREBP-1 was significantly increased with the progression of adipocyte differentiation. AICAR treatment at the concentration of 0.5 mM significantly blocked the expression of these key adipogenic transcription factors (Fig. 3b), further confirming that AICAR inhibits adipocyte differentiation by inhibiting expression of C/EBPα, PPARγ and SREBP-1. Modulation of the expression of other adipocyte-specific markers including Pref-1, aP2 and adipsin, further supported these observations (Fig. 3b). Pref-1 is a marker for preadipocytes and inhibits adipocyte differentiation [31], whereas, aP2 and adipsin are markers for differentiated adipocytes [32,33]. The expression of Pref-1 was significantly reduced with increase in adipocyte differentiation; however, its expression was restored with AICAR treatment (Fig. 3b). AICAR treatment also reduced the expression of aP2 and adipsin mRNA as compared to untreated differentiated cells (Fig. 3). Taken together, AICAR inhibited the expression of key transcription factors (C/EBPα, PPARγ and SREBP-1), adipocyte specific markers, thereby blocking the adipocyte differentiation.

### Adenosine kinase inhibitor inhibits AICAR mediated inhibition of 3T3L1 differentiation

**Figure 4:**
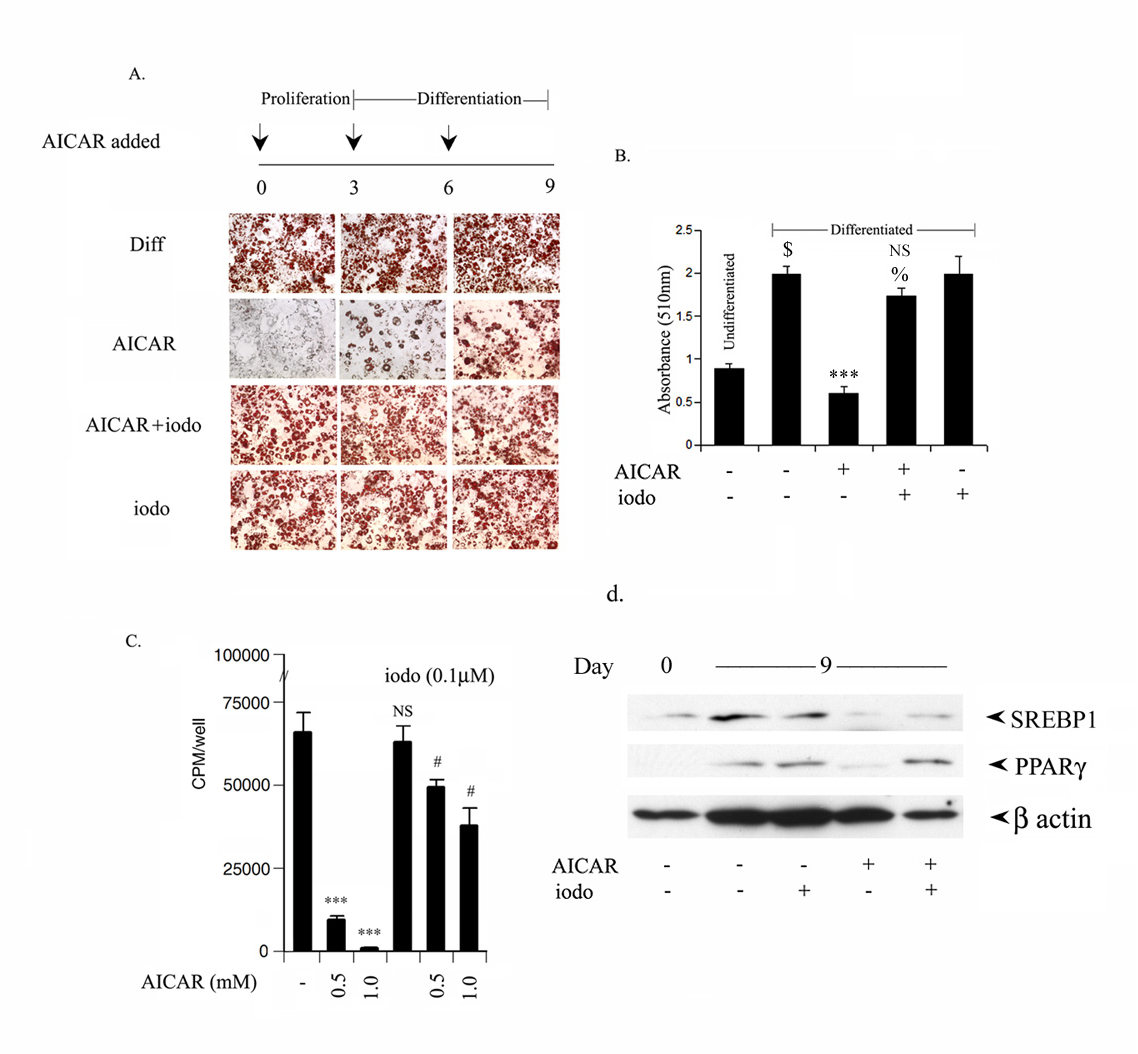
**Adenosine kinase inhibitor (iodotubercidin) reversed the AICAR induced inhibition of 3T3L1 adipocyte differentiation**. The differentiating 3T3-L1 cells were treated with AICAR (0.5 mM) at various differentiating stages (on 0 day, 3^rd ^and 6^th ^day) in the presence or absence of iodotubercidin (0.1 μM). Intracellular lipids were stained with Oil Red O (a). To estimate extent of adipose conversion, 0.2 ml of isopropanol was added to a 12 well plate after 9 days of treatment when AICAR/iodotubercidin was added at 0 day. The extracted dye was immediately removed by gentle pipetting and its optical density was monitored spectrophotometrically at 510 nm (b). Values are mean ± S.D. of three determinations. $ *p *< 0.001 as compared with untreated cells. *** *p *< 0.001 as compared with differentiated cells. NS not significant; % *p *< 0.001 as compared with differentiated cells and AICAR treated cells, respectively. 3T3-L1 cells were treated with AICAR/iodotubercidin (0.5 mM/0.1 μM) during the differentiation induction phase (from day 0 to day2) followed by [3H] thymidine uptake to examine cell proliferation (c). Values are mean ± S.D. of three values. *** *p *< 0.001; NS not significant as compared with untreated cells. * *p *< 0.05, *** *p *< 0.001 as compared to AICAR treated cells (0.5 and 1.0 mM), respectively. 3T3-L1 cells were differentiated in the presence or absence of AICAR/iodotubercidin (0.5 mM/0.1 μM) for 9 days. The protein expression of SREBP1, PPARγ and β actin were examined by immunoblot analysis as described previously (d).

We were further interested in examining the role of ZMP (a phosphorylated form of AICAR, which mimics AMP to activate AMPK) in the regulation of AICAR mediated inhibition of adipocyte differentiation. 3T3L1 cells were treated with AICAR (0.5 mM) on different days (from 0, 3 and 6^th ^day) in the presence or absence of iodotubercidin. Iodotubercidin is an inhibitor of adenosine kinase, and inhibits the conversion of AICAR to its activated form ZMP inside the cell, and thus inhibits activation of AMPK by AICAR [28,34]. Treatment with AICAR at any stage on either day 0 (proliferation phase), day 3 (differentiation phase) or day 6 (late differentiation phase), significantly blocked the accumulation of neutral lipid content as evident from Oil Red staining (Fig. 4a). Addition of iodotubercidin together with AICAR at different stages (day 0, 3^rd ^or 6th) completely abolished the AICAR mediated inhibition of neutral lipid content accumulation (Fig. 4a), while iodotubercidin treatment alone had no effect on adipocyte differentiation. These results were further confirmed by examining for total lipid staining where differentiated 3T3L1 cells have high levels of lipids, which was completely abolished by AICAR treatment (Fig. 4b). Addition of iodotubercidin significantly reversed AICAR mediated inhibition in total lipid content (Fig. 4b). Moreover, iodotubercidin also reversed the AICAR induced inhibition of cell proliferation in 3T3L1 cells as well as the expression of SREBP1 and PPARg  (Fig. 4c, 4d), further suggesting the involvement of ZMP in AICAR mediated inhibition of adipocyte differentiation of 3T3L1.

**Figure 5:**
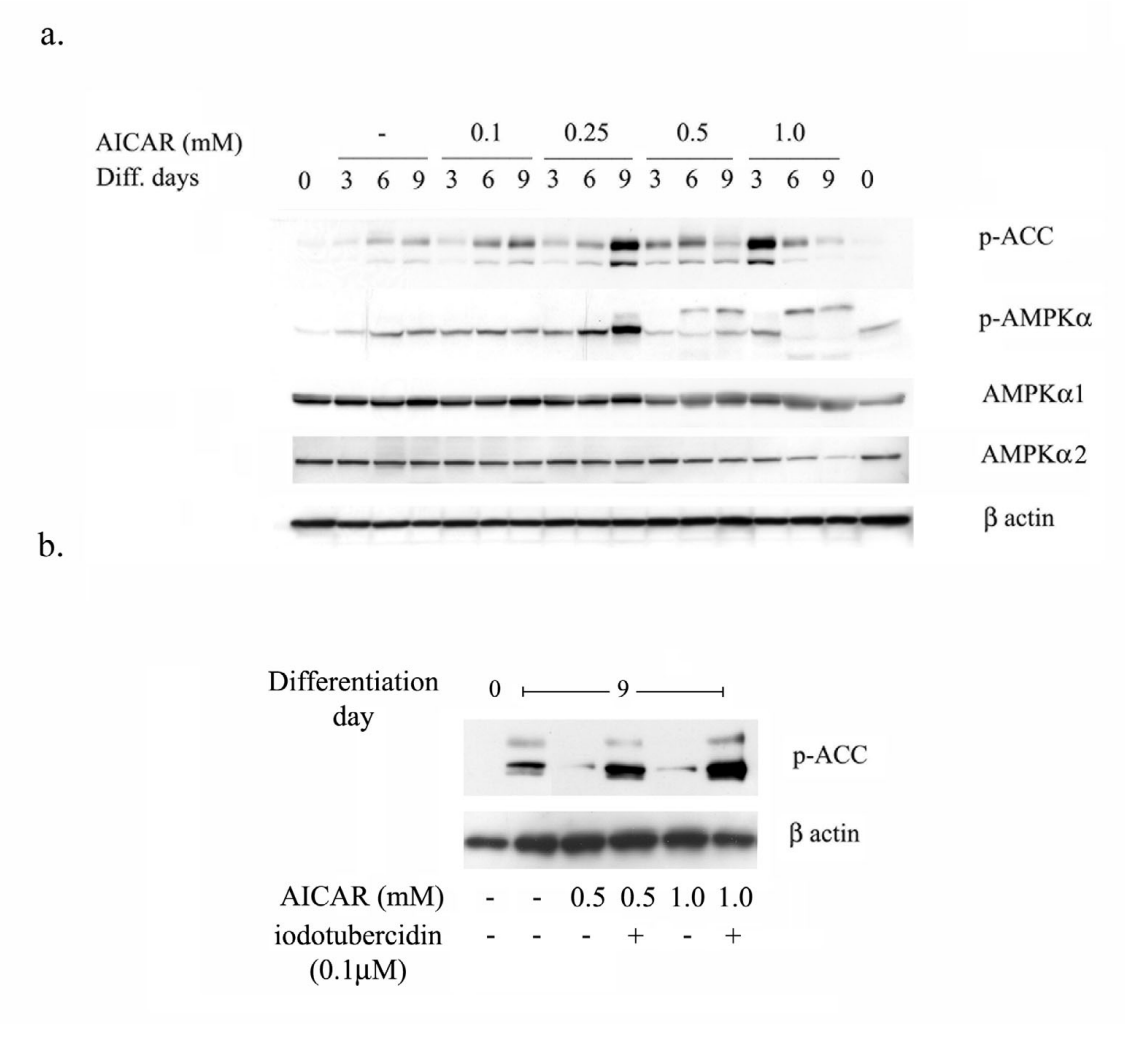
**Effect of AICAR on phospho-status of ACC, AMPK during adipocyte differentiation**. The differentiating 3T3-L1 cells were treated with different concentrations of AICAR (from 0.1 to 1.0 mM) and proteins were isolated at various time periods (from 0 to 9 days). Immunoblot analysis for p-ACC, p-AMPKα, AMPKα1, AMPKα2 and β actin was examined as described in Materials and Methods (a). 3T3L1 cells were differentiated as above in the presence or absence of AICAR (0.5 and 1.0 mM)/iodotubercidin (0.1 μM) for 9 days. Cells were processed for the immunoblot analysis for p-ACC and β actin (b).

Since ZMP is known to mimic AMP and activates AMPK, the effect of AICAR on the phosphorylation status of AMPK and ACC (acetyl CoA carboxylase) at various time points was examined. As shown in figure 5a, the adipocyte differentiation progressed with time; the phosphorylation status of ACC and AMPK also increased, suggesting that activation of AMPK occurs during adipocyte differentiation. Treatment with AICAR further significantly increased the phospho-status of ACC and AMPK in a dose dependent manner, suggesting that AICAR induce the activation of AMPK (Fig. 5).

To investigate whether the activation of AMPK is important or not for adipocyte differentiation, cells were treated with iodotubercidin together with AICAR for 9 days. Chronic treatment with AICAR reduced the levels of phospho-status of ACC, which was restored by iodotubercidin treatment (Fig. 5b).

### AICAR differentially regulates body weight in Diet induced obesity (DIO)

**Figure 6:**
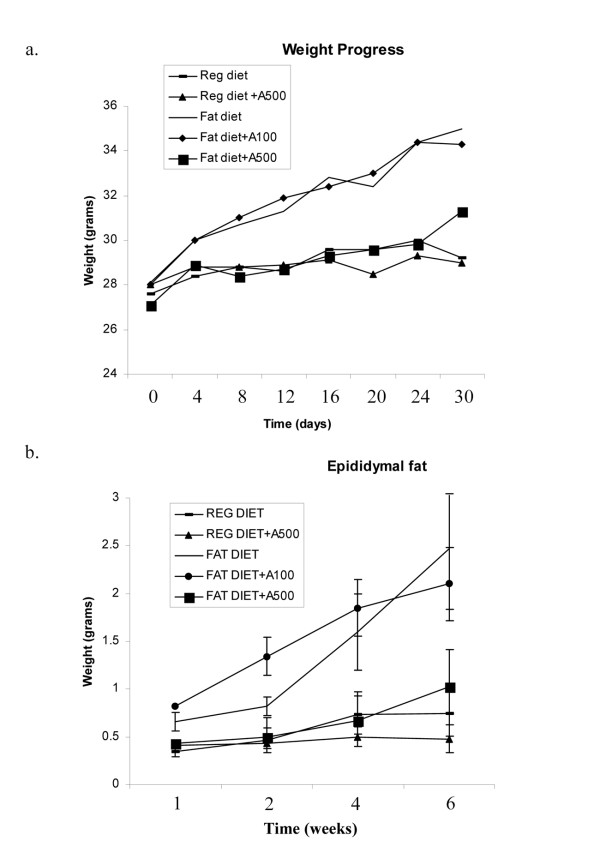
**Effect of AICAR on body weight and epididymal fat content in STD and diet induced obesity mice model**. C57B6 mice were kept on standard (STD) or 60% fat diet (DIO) treated with AICAR (0.1 or 0.5 mg/g body weight) or PBS for 6 weeks intraperitoneally. Body weight of these mice with AICAR was measured periodically (a). At the indicated time periods, epididymal fat was isolated and weighed (b). Results are shown as mean ± SD of 10 mice.

To assess whether AICAR can affect adipocyte formation in vivo, we used the diet induced obesity (DIO) model. C57BL/6J mice were fed either a standard diet (STD) or a high-fat diet (DIO) for 6 wk beginning at 10 wk of age, and treated with AICAR (3 times in a week) at 0.1 mg or 0.5 mg/g body weight for 6 wk. At the beginning of treatment with AICAR, DIO mice weighed 36.1 ± 3.0 g, while STD mice weighed 28.7 ± 2.5 g. The difference between the 2 groups was 21% (Fig. 6a). In the DIO group, the body weight markedly changed following 1 wk treatment, where the difference between AICAR-treated and vehicle-treated animals reached -8.7%. The body weight was then maintained in AICAR-treated mice, while it constantly increased in the vehicle treated group of DIO mice. At the end of the experiment (6 weeks), AICAR-treated DIO mice weighed 15.7% less (-5.7 g) than vehicle-treated DIO mice. In the STD group, AICAR-treated animal weights were not significantly changed. However, AICAR at a dose of 0.1 mg/g bw had no effect on body mass, as compared to DIO vehicle treated mice. In accordance, DIO vehicle treated mice had significantly higher epididymal fat content as compared to STD mice (Fig. 6b). AICAR (0.5 mg/g bw) treated DIO mice had a significant reduction in epididymal fat content on par with STD mice, however, AICAR at the dose of 0.1 g/mg bw had no effect (Fig. 6b). These results indicate that the higher body weight detected in DIO vehicle treated mice was at least in part due to an increase in adipose tissue mass, and that AICAR administration (0.5 mg/g bw) significantly normalized the body weight as well as the fat content (adipose tissue mass) similar to those in STD vehicle treated mice.

### AICAR inhibits adipocyte size without modulating cell number or adipogenesis in DIO-treated mice

**Figure 7:**
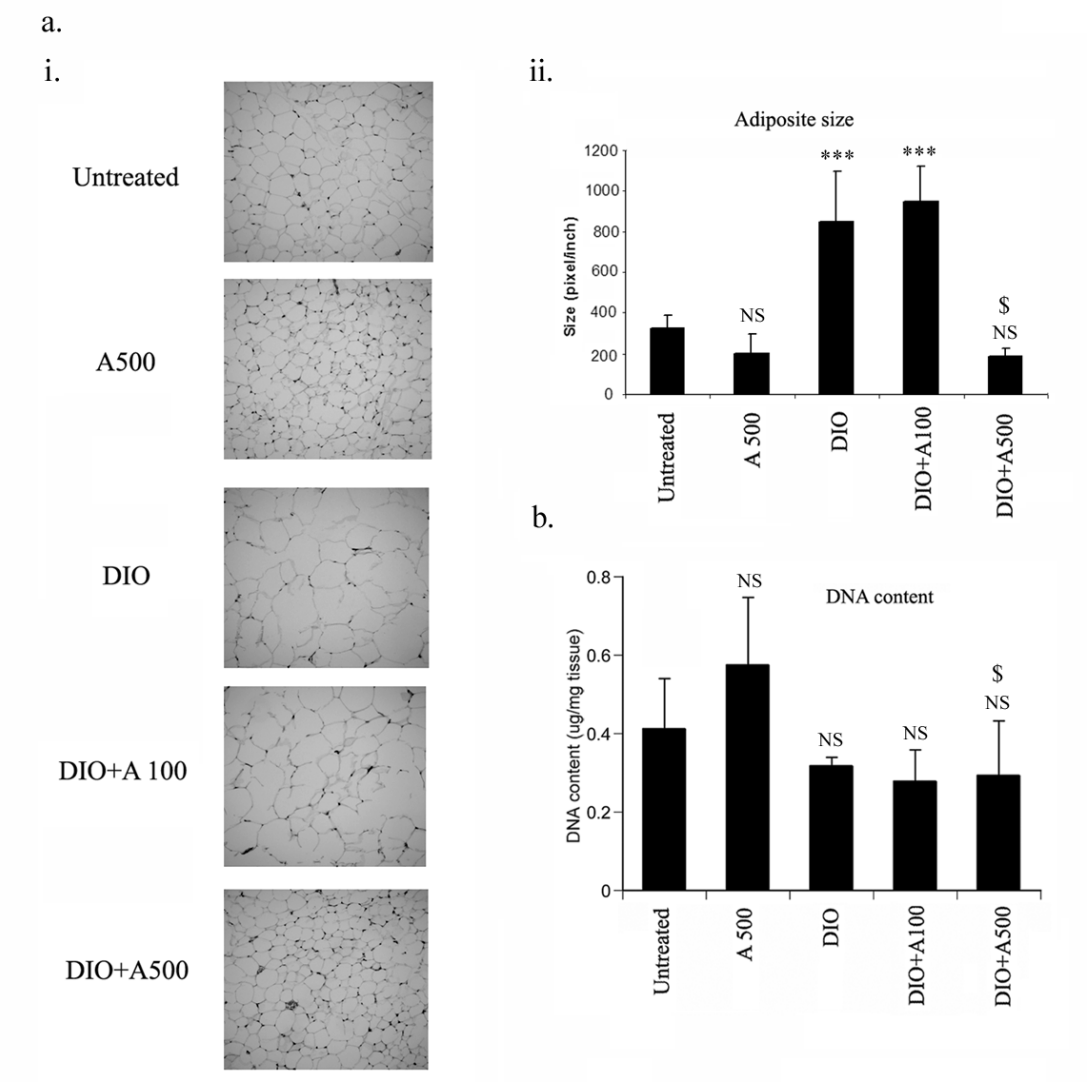
**Effect of AICAR on adipocyte size and total DNA content in AICAR treated STD and DIO mice**. Paraffin-embedded sections of epididymal white adipose tissue from AICAR treated STD and DIO mice were stained with hematoxylin and eosin (ai). Average size of adipocytes measured in 10 different fields is shown at *right *(aii). ***p < 0.001; NS: not significant as compared to STD mice group. $ *p *< 0.001, as compared to DIO vehicle treated group. NS: not significant as compared to STD mice group. White adipose tissue from these treated STD and DIO mice were digested with proteinase K and DNA was extracted with phenol:chloroform. DNA content was measured by spectrophotometer (260 nm) (b). NS: not significant as compared to STD mice group. $: not significant as compared to DIO vehicle treated group.

Since AICAR administration restricted body weight as a consequence of reduction in fat content of DIO mice, it was of interest to examine whether AICAR treatment controlled the adipose size or restricted the number of cells. The size of adipocytes were ascertained by microscopic analysis and it was observed that DIO vehicle treated mice had a significant increase in adipocyte size, which was significantly restricted in AICAR treated mice at the dose 0.5 mg/g bw while there was no effect at 0.1 mg/g bw (Fig. 7a). On the other hand, no change in the total DNA content in the adipose tissue of the AICAR treated and vehicle treated DIO mice was observed, indicating that cell number was maintained in both cases (Fig. 7b). This suggested that the decrease in epididymal adipose tissue in AICAR treated DIO mice as compared to DIO vehicle treated mice was not due to a decrease in cell number.

### Effect of AICAR on adipogenic markers in STD and DIO treated mice

**Figure 8:**
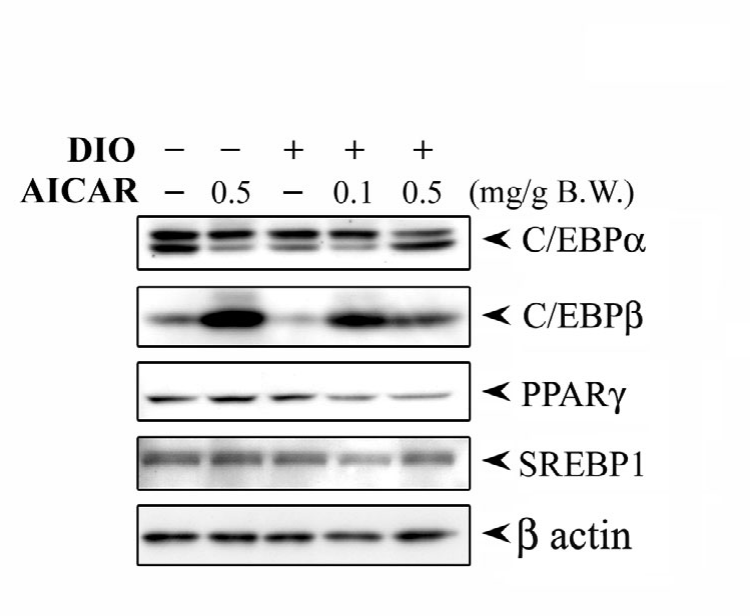
**Effect of AICAR on adipocyte protein marker expression in DIO treated mice after 6 weeks of treatment**. Total protein epididymal fat content from 6 weeks AICAR treated STD and DIO treated mice was isolated. The expression for various key adipogenic transcription factors including C/EBPα, C/EBPβ, PPARγ and SREBP1 was examined by immunoblot analysis as described in Materials and Methods.

To examine further whether the decrease in epididymal fat content was due to an increase in the adipocyte differentiation process, the expression levels for several adipocyte markers were examined by immunoblot analysis. As shown in Fig. 8a, the decrease in epididymal fat content observed in AICAR treated mice (0.5 mg/g bw) was not reflected in the change in the expression of adipocyte marker genes such as C/EBPα, C/EBPβ, PPARγ, and SREBP1. However, we observed a significant increase in the expression of DGAT and mitochondrial GPAT, two enzymes involved in the synthesis of triglycerides, by AICAR treatment in STD mice as well as in DIO treated mice (data not shown).

**Figure 9:**
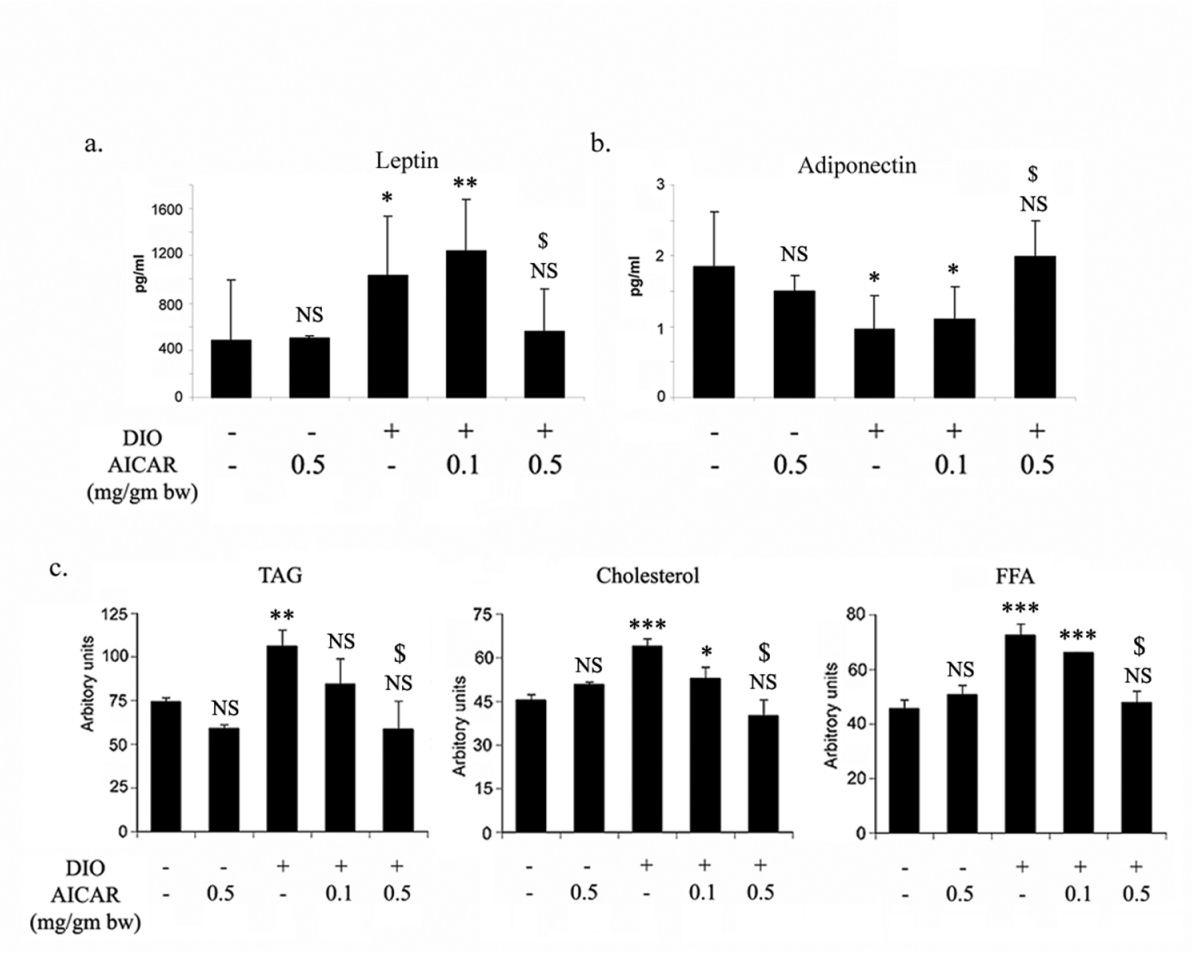
**Effect of AICAR on metabolic alterations in AICAR treated STD and DIO treated mice**. Blood was withdrawn from AICAR treated STD and DIO treated mice after 6 weeks of treatment and serum was isolated and the levels of leptin and adiponectin were examined by the respective ELISA kits as described in Material and Methods (a). Values are mean ± S.D. of eight values. * *p *< 0.05; ** *p *< 0.01; NS: not significant as compared to STD mice group. $ *p *< 0.05, as compared to DIO vehicle treated group. NS: not significant as compared to STD mice group. The lipid profile (TAG, cholesterol and FFA) was examined as described in Material and Methods (b). Values are mean ± SD of 4 different sera. For TAG, ** *p *< 0.01; NS: not significant as compared to STD mice group. $ *p *< 0.001, as compared to DIO vehicle treated group. NS: not significant as compared to STD mice group. For Cholesterol, *** *p *< 0.001; * *p *< 0.05; NS: not significant as compared to STD mice group. $ *p *< 0.001, as compared to DIO vehicle treated group. NS: not significant as compared to STD mice group. For FFA, *** *p *< 0.001; NS: not significant as compared to STD mice group. $ *p *< 0.001, as compared to DIO vehicle treated group. NS: not significant as compared to STD mice group.

Biologically active molecules secreted from adipose tissue called adipokines, have been implicated in the development of obesity. The levels of leptin are significantly increased with the increase in adipose tissue content in DIO vehicle treated mice. However, AICAR (0.5 mg/g bw) treatment for 6 weeks brought these back to the normal levels on par with STD mice (Fig. 9a). In contrast to leptin levels, adiponectin levels were drastically reduced in DIO vehicle mice, which also normalized with AICAR treatment (Fig. 9b). Moreover, DIO mice exhibited pronounced dyslipidemia compared to STD mice, as evident from the elevated levels of triglyceride (TAG), cholesterol and free fatty acids (FFA), which were significantly reduced by chronic treatment of AICAR (0.5 mg/g bw) (Fig. 9c). Furthermore, diet-induced increase in saturated fatty acids (16:0, 18:0) and decrease in major polyunsaturated fatty acid (18:2) in adipose tissue were also normalized by chronic AICAR treatment (Table 1).

**Table 1:** Effects of AICAR treatment on fatty acid composition (weight %) of adipose tissue in normal and diet-induced obese mice

**Group/Fatty acid**	**14:0**	**16:0**	**18:0**	**18:2**
**STD**	0.89 ± 0.02	18.6 ± 0.1	1.9 ± 0.06	27.1 ± 0.35
**STD+A500**	0.9 ± 0.01	22.2 ± 0.21	3.0 ± 0.15	28.9 ± 0.81
**DIO**	1.3 ± 0.2	26.6 ± 0.21*	3.6 ± 0.15*	15.9 ± 1.3*
**DIO+A100**	0.95 ± 0.03	24.3 ± 0.38	4.4 ± 0.06	28.7 ± 0.86
**DIO+A500**	1.1 ± 0.1	23.3 ± 0.26**	2.5 ± 0.21**	22.2 ± 0.7**

Adipose tissue was isolated and prepared as described in Methods. The lipids were extracted in chloroform-methanol using Folch partition. Fatty acid methyl ester was prepared with methanolic-HCl, purified by TLC and analyzed by GC as described in our earlier publication [29]. Fatty acids showing significant alteration in composition are included and their peaks were identified using known standards. Values are expressed as mean ± SD for 3 separate sets of experiments. *p < 0.001 vs STD, **p < 0.001 vs DIO.

**Figure 10:**
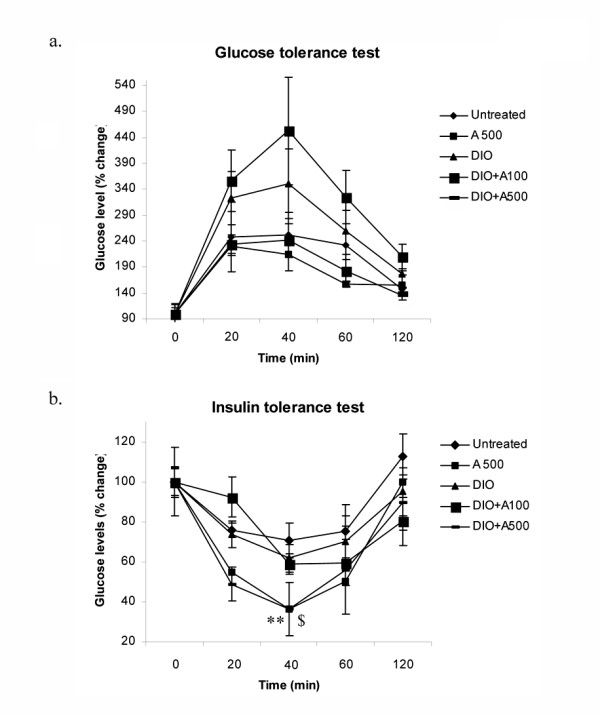
**Effect of AICAR on glucose and insulin tolerance tests in AICAR treated STD and DIO treated mice**. For glucose tolerance test, overnight-fasted mice from all groups were given an intraperitoneal injection of glucose (2 mg/g body wt). Blood samples were withdrawn from the tail and analyzed for glucose levels at the indicated time points (15 to 120 min) (a). For insulin tolerance test, insulin (0.5 units/kg body wt) was intraperitoneal injected after fasting for 12 h, and glucose levels were monitored at the indicated time points (15 to 120 min)(b). Results are the means ± SD from four animals.

Lipid accumulation and alterations in fatty acid composition in various tissues as a result of obesity is considered to be a cause of development of insulin resistance. Therefore, we examined the glucose tolerance test and insulin tolerance test in DIO-vehicle and AICAR treated mice after 6 weeks of treatment. As depicted from figure 10a &10b, DIO-vehicle and AICAR treatment at a dose of 0.1 mg/g bw showed hyperglycemia and hyperisulinemia, however, AICAR treated DIO mice at the dose of 0.5 mg/g bw completely ameliorated hyperglycemia and hyperisulinemia induced by the high fat diet. We also determined the TGA and FFA content in liver of DIO vehicle and AICAR treated DIO (0.5 mg/g bw) mice and observed that DIO vehicle treated mice exhibited a significant increase in TGA and FFA levels, which were significantly inhibited by AICAR treatment (data not shown). This indicates that the differences in glucose tolerance and insulin sensitivity observed between DIO and AICAR treated DIO (0.5 mg/g bw) mice is due to a decrease in lipid storage in adipose tissue and liver in AICAR treated DIO mice (0.5 mg/g bw), which probably prevented the further impairment of glucose homeostasis. Since lipid accumulation in tissues other than adipose tissue, such as liver, is considered to be a cause of the development of insulin resistance associated with obesity.

### AICAR activates AMPK in adipose tissue

**Figure 11:**
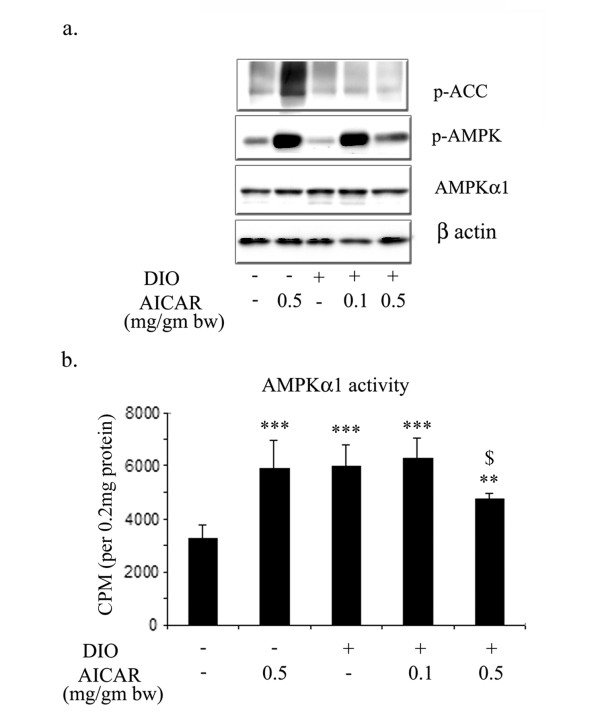
**Effect of AICAR on phospho-status of ACC, AMPK and AMPK enzymatic activity in AICAR treated STD and DIO treated mice**. Total protein from epididymal fat content from 6 weeks AICAR treated STD and DIO treated mice was isolated. Immunoblot analysis for p-ACC, p-AMPKα, AMPKα1 and β actin was examined as described in Materials and Methods (a). The enzymatic activity of AMPKα1 was examined by immunoprecipitating using anti-AMPKα1 followed by assessing its activity using SAMS peptide as described in Material and methods (b). *** *p *< 0.001; ** *p *< 0.01 as compared to STD mice group. $ *p *< 0.05, as compared to DIO vehicle treated group.

We examined further the possibility of activation of AMPK in adipose tissue by chronic treatment with AICAR. For this, we examined the phospho-status of AMPKα and ACC as well as the total level of AMPKα1 in adipose tissue after 6 weeks of AICAR treatment in STD and DIO mice. AICAR treatment at both doses induced the phosphorylation of AMPKα as compared to STD and DIO vehicle treated mice (Fig. 11a). However, the phospho-status of ACC was only increased in the AICAR treated STD mice, and did not show any change in DIO-vehicle treated, as well as in AICAR treated DIO mice. No change was observed in the total level of AMPKα1 in any treatment in STD and DIO mice. However, a significant increase in the enzymatic activity of AMPKα1 was observed in AICAR treated STD, in DIO-vehicle treated and AICAR treated (0.1 mg/g bw) adipose tissue (Figure 11b). AICAR treated adipose tissue at a dose of 0.5 mg/g bw also showed a significant increase in AMPKα1 activity but the increase in its activity was not significantly higher than that observed in other group.

### AICAR induced expression of PGC1α

**Figure 12:**
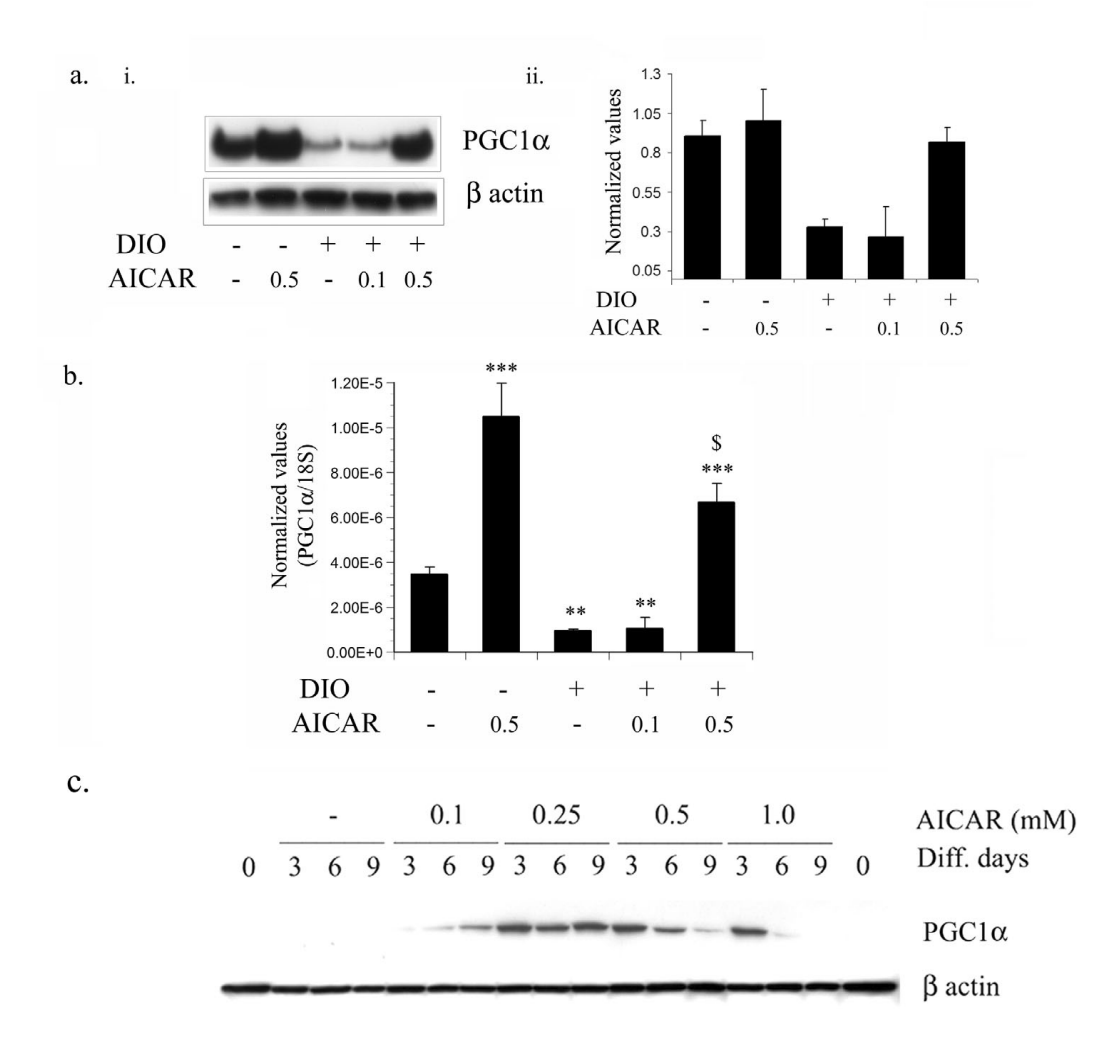
**AICAR induced the expression of PGC1α in white adipose tissue in DIO treated mice**. Total protein from epididymal fat content from 6 weeks AICAR treated STD and DIO treated mice was isolated and immunoblot analysis for PGC1α was examined (a i). Densitometry values for PGC1α were normalized with β actin and plotted as a bar graph (a ii). The expression of PGC1α was quantified by qPCR (b). The relative qPCR values were normalized with respect to the 18S rRNA expression level. Values are mean ± S.D. for three mice. *** *p *< 0.001; ** *p *< 0.01 as compared to STD mice group. $ *p *< 0.001, as compared to DIO vehicle treated group. The differentiating 3T3-L1 cells were treated with different concentrations of AICAR (from 0.1 to 1.0 mM) and proteins were isolated at various time periods (from 0 to 9 days). Immunoblot analysis for PGC1α and β actin was examined as described in Materials and Methods (c).

Proliferator-activated receptor γ coactivator 1α (PGC-1α) is a coactivator of nuclear receptors and other transcription factors [35]. PGC-1α controls mitochondrial biogenesis and oxidative metabolism in many tissues, including brown adipose tissue, skeletal muscle, heart, and liver [36-39]. We examined the effect of AICAR on DIO vehicle treated mice and observed that AICAR treatment of STD mice significantly induced protein expression as compared to that in untreated STD mice (Fig. 12a). The expression of PGC1α was found to be significantly down in adipose tissue in DIO vehicle and AICAR (0.1 mg/g bw) treated mice, however AICAR at the dose of 0.5 mg/g bw significantly reversed the protein expression of PGC1α induced in DIO as documented by immunoblot and its densitometry analysis (Figure 12ai &12aii). These observations were further supported by quantitative PCR for PGC1α in adipose tissue from AICAR treated STD and DIO mice (Fig. 12b). To examine the effect of AICAR on the expression of PGC1α, 3T3L1 cells were treated with increasing concentrations of AICAR (0.1–1.0 mM) under differentiating condition and the expression of PGC1 α was examined at 3, 6 and 9 days using immunoblot analysis. As depicted from figure 12c, treatment of AICAR induced the protein expression of PGC1α at day 3 in dose dependent manner, however, higher concentration of AICAR (0.5 and 1 mM) induced the highest expression at day 3 after that there was a decrease in its expression.

## Discussion

Obesity is a metabolic disorder, resulting from an imbalance between metabolizable energy intake and energy expenditure [40]. The increase in obesity is particularly marked in children, and not just confined to the Western world [41]. In the present study, we examined the effect of AICAR, a pharmacological activator of AMPK, on *in vitro *adipocyte differentiation of 3T3L1 and *in vivo *diet induced obesity mouse model (DIO). We observed that AICAR inhibited adipocyte differentiation in 3T3L1 cells via downregulation of important transcription factors such as C/EBPα, PPARγ and SREBP1, which strictly control adipocyte differention.

The CCAAT element-binding proteins (C/EBP) β and δ and sterol response element-binding protein 1 (ADD1/SREBP1) are active during the early stages of the differentiation process and induce the expression and/or activity of the peroxisome proliferator-activated receptor γ (PPARγ), a pivotal coordinator of adipocyte differentiation [42]. Activated PPARγ induces exit from the cell cycle, and in cooperation with C/EBPα, stimulates the expression of many metabolic genes [42]. Our observation is in accordance with a previous report where AICAR was shown to inhibit C/EBPα and PPARγ due to the inhibition of early clonal expansion of pre-adipocytes [43]. Our data shows that inhibition of adipogenic differentiation in 3T3L1 is not just confined to early clonal expansion, since addition of AICAR either at early differentiation stage (day 3) or at late differentiation stage (day 6), significantly blocked 3T3L1 differentiation. We speculate this effect is due to the repression of PPARγ dependent transcription by AMPK activation via phosphorylation of p300 at serine 89 [44,45], however, it remains to be established in adipogenic differentiation.

During 3T3L1 differentiation, there was an increase in ACC and AMPK phosphorylation at days 3, 6 and 9, suggesting the activation of AMPK. But the role of activation AMPK in the regulation of differentiation remains to be explored. Incubation of 3T3L1 cells, undergoing differentiation, with AICAR at the concentration of 0.1 and 0.25 mM, induced the phosphorylation of ACC and AMPK at day 6 and 9. However, at the higher concentration (0.5 and 1 mM) it does not induce ACC and AMPK phosphorylation compared to untreated differentiated cells at day 9. Moreover, adenosine kinase inhibitor (iodotubercidin) completely reversed AICAR mediated inhibition of adipocyte differentiation, expression of SREBP-1 and PPARγ. Increase in phosphorylation of ACC and AMPKα during adipocyte differentiation in time dependent manner indicating the possible activation/involvement of AMPK during adipocyte differentiation. Moreover, adenosine kinase inhibitor also reversed the AICAR-mediated downregulation of phosphorylation of ACC at day 9, further suggesting that AICAR mediated effect may not be due to AMPK activation and requires more in-depth studies in this process.

To investigate further the effect of AICAR on adipocyte content *in vivo*, we employed the diet induced obesity model where we administered two doses of AICAR (0.1 and 0.5 mg/g kb) for 6 weeks. The lower dose of AICAR (0.1 mg/g) did not modulate either the body weight or total epididymal fat content in DIO treated mice. However at 0.5 mg/g bw dose, AICAR was found to be very effective in maintaining body weight and epididymal fat content, restoring levels of adipokines and significantly improving glucose tolerance and insulin sensitivity, as compared to DIO vehicle treated mice. Other reports have demonstrated chronic administration of AICAR to diminish the mass of fat pads significantly while having no effect on body weight in treated rats [24,46]. Another report [47] showed that in fatty rats (fa/fa) treated with AICAR (0.5 mg/g) daily resulted in a modest decrease in intra-abdominal adiposity (15%), as compared with pair-fed animals and improved metabolic disturbances. All these reports suggest that AICAR administration leads to decrease in food intake [24,46,47], however, we did not observe any change in food intake at any dose of AICAR treatment (Data not shown). AICAR treatment (0.5 mg/kg) also restored the levels of adipokines (adiponectin and leptin) in DIO mice as compared to vehicle treated mice. The main sources of these adipokines, the adipose tissue, are well known to activate AMPK and it will be interesting to understand how AICAR regulates the production of these adipokines in DIO treated mice. In contrast to our *in vivo *observation, AMPK activators have been shown to inhibit the production of adiponectin in 3T3L1 adipocytes [48].

AICAR administration induced the activation of AMPKα1 enzymatic activity as well as phosphorylation of AMPKα in STD and DIO treated adipose tissue. However, adipose tissue from DIO vehicle treated mice showed significant activation of AMPKα1 as compared to adipose tissues from STD vehicle treated mice. This is in contrast with another report, where they did not observe any changes in the AMPK activity in adipose tissue treated with AICAR, even though the accumulation of ZMP was observed in adipose tissue of these animals [47]. The molecular mechanisms underlying the role of AMPK in adipose tissue are not fully known. Adipose tissue from 0.1 mg/kb AICAR treated DIO mice also exhibited increase AMPKα1 activity. However, there is no reduction in metabolic alteration compared with untreated DIO mice further questioning the involvement of AMPK in the regulation of metabolic alteration.

Increase in adipose tissue mass may be due to an increase in the number of adipose cells, or an increase in triglyceride storage in the preexisting adipocytes, or a combination of both processes. Though AICAR administration significantly decreased epididymal fat content, no change was observed in the expression of key adipogenic factors such as C/EBPα, PPARγ, SREBP-1 with significant induction of C/EBPβ in AICAR treated STD and DIO treated animals. Untreated STD animals had significant expression of these adipogenic factors suggesting that STD mice already had fully differentiated adipocytes, which suggests that the reduction in epididymal fat content observed in AICAR treated DIO mice is not due to blockage of preadipocyte differentiation into adipocytes as observed *in vitro *3T3L1 adipocyte differentiating model. We observed the reduction of C/EBPβ levels in DIO vehicle treated mice, which is restored by AICAR treatment. The expression of C/EBPβ decreased as adipocyte differentiation progressed. Restoration of C/EBPβ protein by AICAR treatment can be due to the suppression of cytoplasmic HuR protein [49], which may result in the accumulation of C/EBPβ protein since C/EBPβ message is a ligand for HuR [50] and regulates the early event of adipogenesis.

Another possibility for the reduction of epididymal fat content and the size of adipocytes observed in AICAR treated DIO mice may be due to cellular hypotrophy which results in decreased triglyceride accumulation inthe pre-existing adipocytes, rather than an increase in cell number or differentiation. In AICAR treated DIO mice, the decreasedtriglyceride accumulation in adipose tissue could be due to decreased triglyceride synthesis or increased lipolysis. Triglyceride synthesis is regulated by mitochondrial GPAT and DGAT, two enzymes involved in the esterification of fatty acids to triglycerides. Mitochondrial GPAT have been shown to be the substrates for AMPK, resulting in the inhibition of enzymatic activity [51]. According to our observation AICAR treatment (0.5 mg/g bw) significantly induced the expression of both GPAT and DGAT in adipose tissue of STD and DIO treated animals. The decrease in the content of adipose tissue in AICAR (0.5 mg/g bw) treated DIO and increase in the expression of these enzymes cannot be explained at present. In terms of regulation of lipolysis in rodent adipocytes, the role of AMPK remains controversial [52,53]. Recently, using pharmacological activators, adenoviruses expressing either dominant negative or constitutively active forms of AMPK and AMPKα1 knockout mice, have clearly demonstrated that AMPK activation has an anti-lipolytic effect in rodent adipocytes [54]. At present, we do not have any explanation how AICAR reduced the epididymal fat content in DIO treated mice when the expression of GPAT and DGAT is significantly increased and AMPK has been shown to be anti-lipolytic in adipocytes [54].

Decrease in adiposity caused by AICAR in DIO treated mice is attributable, at least in part, to increase in energy expenditure. AICAR has been shown to increase expression of the uncoupling proteins UCP3 [55,56] and the transcription factor PGC1α [57] in rat skeletal muscle. Peroxisomal proliferator activator receptor (PPAR) gamma co-activator 1alpha (PGC1α) is a transcriptional coactivator that has recently been highlighted as an important regulator of gluconeogenesis, fatty-acid oxidation and adaptive thermogenesis [37]. Moreover, the expression of PGC1α was found to be reduced in skeletal muscle of prediabetic and diabetic subjects [58] and in adipose tissue of morbidly obese people [59]. We also observed the reduced protein and mRNA expression of PGC1α in adipose tissue of DIO vehicle treated mice, which was significantly induced by AICAR treatment not only in DIO treated mice but also in STD treated mice. Similar observation was found in 3T3L1 cells when cells were treated with different concentration of AICAR, the expression of PGC1α increased in dose dependent manner at day 3. Why treatment of of higher concentration of AICAR (0.5 and 1 mM) abolished the expression of PGC1α at days (6 and 9), which was induced at day 3, can not be explained at present? However, AICAR mediated reduction in adipose tissue of DIO treated mice may be due to the expression of PGC1α, which plays an important role in adaptive thermogenesis by regulating the expression of UCP-1, a key molecule that uncouples ATP synthesis from mitochondrial respiration, resulting in heat production [35]. Recent two studies also supported our observation that AICAR treatment induces the PGC1α expression [60,61]. Moreover, AMPK has also been shown to induce mitochondrial biogenesis [60] and up-regulate nuclear respiratory factor-1 (NRF-1) whose expression is controlled by PGC1α [62].

## Conclusion

Our study demonstrates that AICAR treatment significantly attenuates adipoctye differentiation *in vitro*. However, its administration restricted the body weight, epididymal fat content and normalized metabolic alteration mediated by diet induced obesity in mice. Increase in phosphorylation of ACC and AMPKα during adipocyte differentiation and AMPK activity in epididymal adipose tissue in DIO mice raises doubts about the involvement of AMPK in this process.

## Abbreviations

AICAR (5-aminoimidazole-4-carboxamide-1-beta-4-ribofuranoside); DIO Diet induced obesity; PPARγ peroxisome proliferators-activated receptor γ; C/EBP CCAAT/enhancer binding protein; ADD1 adipocyte differentiation and determination factor 1; SREBP1c sterol regulatory element binding protein 1c; TNFα tumor necrosis factor-α; IL-6 interleukin-6; WAT white adipose tissue; AMPK AMP-activated protein kinase; AMPKK AMP-activated protein kinase kinase; ACC acetyl CoA carboxylase; PGC1α Proliferator-activated receptor γ coactivator 1α; NRF-1 nuclear respiratory factor-1; SDS-PAGE (sodium dodecyl sulfate-polyacrylamide gel electrophoresis);

## Competing interests

The author(s) declare that they have no competing interests.

## Authors' contributions

SG carried out the various experiments, participated in the design of the study and drafted the manuscript; RR and EH performed animals and biochemical studies; RR also helped in drafting manuscript; MK and RY performed lipid studies; J-S W helped in performing biochemical assays and immunoblot analysis; LK, AKS and IS participated in the design of the study and helped to draft the manuscript. All authors read and approved the final manuscript.
